# Intelligent Transportation Logistics Optimal Warehouse Location Method Based on Internet of Things and Blockchain Technology

**DOI:** 10.3390/s22041544

**Published:** 2022-02-17

**Authors:** Jun Chen, Shiyan Xu, Kaikai Liu, Shuqi Yao, Xiao Luo, Huan Wu

**Affiliations:** 1 SILC Business School, Shanghai University, Shanghai 201899, China; chenjun1@shu.edu.cn (J.C.); kaikailiu@shu.edu.cn (K.L.); yaoshuqi2019@shu.edu.cn (S.Y.); luoxiao2002@shu.edu.cn (X.L.); wuhuan@shu.edu.cn (H.W.); 2 Economics and Management School, Tongji University, Shanghai 201804, China

**Keywords:** intelligent transportation logistics, optimal warehouse location, IoT, blockchain

## Abstract

In order to cut down on costs to the greatest possible extent, enterprises hope to distribute goods to different customers at the lowest costs possible. Based on this, this paper proposes an optimal location method for an intelligent transportation logistics warehouse. This scheme combines a variety of complex mechanisms to allow IoT devices to provide input. The scheme makes full use of the irreducibility of a blockchain system to promote the development and design of blockchain logistics applications. This method is aimed at tracking the progress of transportation of products in the whole supply chain. Experimental results show that, compared with traditional methods, the optimal positioning method has the advantages of fewer calculations, a high positioning accuracy, and a low overall cost, and it obtains the best warehouse positioning results. Based on the Internet of Things and blockchain technology, the application of intelligent logistics systems enables enterprises to intuitively understand their current inventory and the transportation status of goods, thus better controlling changes in enterprise resources.

## 1. Introduction

Logistics refers to the business of maintaining and managing a commodity supply chain from the output end to the consumption end [1]. Every time that a handover to a new holder occurs, data interactions must be carried out in the form of documents, certificates, contracts, or other important forms [2]. With the rapid growth of the commodity economy, and the continuous cultivation of online shopping habits, the market scope is growing wider while the quantity of goods is increasing. Nowadays, the demand for logistics is increasing sharply, which creates higher requirements in the development of the logistics industry [3]. Because of the popularity of online shopping in the new era, the logistics industry has been booming. Warehousing has become an important part of the supply chain of modern enterprises, and it has developed from original goods storage to other operations, such as transportation, storage, and measurement, involving more and more industries and fields [4]. Intelligent transportation logistics are gradually being widely used by large enterprises. In order to save on cost to the greatest extent possible, enterprises hope to distribute goods to different customers for the lowest cost, so it is necessary to study a reliable intelligent logistics warehouse-location methods [5]. The location of customer nodes and customer demand are known and estimable. Goods are supplied by one or more facilities and each customer only receives goods from one facility. The location of potential facilities is known. A goal to address this problem is establishing potential facilities to minimize total costs [6].

At present, the development of China’s logistics industry has started focusing on the field of intelligence, and all major domestic logistics fields are exploring application solutions of the Internet of Things (IoT) in logistics intelligence [7]. IoT-divergent large-scale expansion is the current development trend, and is based on the continuous development and improvement of blockchain technology [8]. For an indoor environment with complex conditions, due to the influence of buildings and complex indoor environments, a signal received by an indoor positioning terminal is very weak. This results in a great decline in positioning accuracy, which cannot meet the accuracy requirements of indoor positioning [9]. The traditional positioning method cannot guarantee both a high and low positioning accuracy at the same time, and the optimal warehouse positioning result is not produced at the lowest cost [10]. Therefore, based on IoT blockchain technology, an optimal warehouse location method for intelligent transportation logistics is proposed. This scheme combines a variety of complex mechanisms, allows IoT equipment input, makes full use of the fact that blockchain systems cannot be altered, and promotes the design and development of a logistics application for blockchain, so as to track the transportation journey of products throughout the whole supply chain process. This scheme aims at realizing mutual trust, transparency, and data integrity between cooperative logistics entities, as well as between logistics entities and customers.

Modern industrial warehouses are often large-scale, and efficiency can be improved by improving accurate warehouse management [11]. In order to better realize practical application of IoT in the contemporary intelligent logistics application field, it is necessary that research on IoT should not remain focused on the connection between materials and the mesh composition of devices, and research on different facets of IoT’s control technology, security system protection technology, data analysis, mining technology, architecture model, etc., be performed In the development and construction of IoT, the role of blockchain cannot be ignored [12]. Blockchain + big data solutions have effectively solved several fundamental problems existing in the IoT logistics application industry, namely, the expansion in the number of users, improvements to the infrastructure, and improved the convenience of transactions [13]. Effective solution of these problems has laid a solid theoretical and practical foundation for application of IoT in the development of logistics intelligence. The traditional method manages a warehouse through video monitoring, which requires real-time monitoring, uses a great deal of manpower, and experiences inevitable mistakes. At the same time, process and loading errors can easily occur in the process of material transportation, and these errors cannot be found in time [14]. Compared with the traditional method, the proposed method has the advantages of low computational requirements, a high positioning accuracy, and a low overall cost. The use of intelligent logistics system based on IoT enables enterprises to have an intuitive understanding of their current inventory, transportation status and other information regarding goods, and can better control changes in enterprise resources. Users can also easily query real-time information about goods in various intuitive ways, which increases the trustworthiness of logistics enterprises.

In the Section 2 of this paper, by the previous research contributions, an optimal warehouse positioning method is put forward to improve its quality. In the Section 3, the basic concept of a warehouse management system based on IoT positioning technology is expounded. In the Section 4, an optimal warehouse positioning method using intelligent transportation logistics, based on IoT and blockchain technology, is proposed, and the optimal warehouse positioning model using intelligent transportation logistics is constructed. Finally, in the Section 5, the model is tested to verify the effectiveness of the method.

## 2. Literature Review

The degree of warehouse management directly determines developmental prospects of an enterprise. In addition, how to reasonably improve operational efficiency and the safety of warehouses is one of the most important considerations of enterprises. However, there are few studies focusing on the use of blockchain technology in logistics, and most of these focus on proposals and improvements to concepts. In [15], the development status and prospects of blockchain solutions for logistics were analyzed from the industrial point of view, and it was noted that many enterprises have already made significant investments in this area, especially in blockchain applications supporting tracing and financial/payment processing. In [16], a fuzzy Petri net was used as a tool to build an analysis model of influencing factors in an aviation logistics blockchain, and the main factors influencing the restriction of the applying aviation logistics blockchain technology was explored. In [17], a blockchain for product traceability using a fast-response coding label was put forward, and the concept of applying blockchain to logistics was explored. In [18], and in other works, the stochastic LRP model was studied, in which the demand for vehicles was only known when they reached customers. The work in [19] considered the problems of facility location, vehicle allocation, and route selection by emergency services at random times. In [20], a mathematical model addressing the positioning–transportation route problem with multiple warehouses, multiple vehicles, and random demands was established, and the mathematical method for solving it was given. In [21], three different architecture methods for application design for IoT devices, based on an Ethereum blockchain, was presented, and it noted that a feasible solution, at present, is to connect IoT devices to a blockchain through gateway units.

In recent years, cloud warehouse mode has emerged in the field of e-commerce logistics, and has shown strong circulation capacity. Based on warehouse distribution networks and advanced software and hardware facilities, the cloud warehouse mode takes big data technology as its core, providing customers with efficient storage, distribution, and a series of value-added services. The author of [22] introduced a composition of the cloud warehouse mode and made suggestions for the development of countermeasures. The authors of [23] stated that, under the new retail mode of order management, “cloud warehouse” storage and other functions of a management system can perfectly realize “decentralized sales, centralized management”. The authors of [24] point out that, after the generation of a new cloud warehouse model, the construction of an intelligent storage system needs to be completed with the support of big data and artificial intelligence, which promotes an integration process for warehouse distribution. This is conducive to a more reasonable use of social resources.

With the rapid development of online shopping, consumers prefer more and more people-oriented services from the logistics industry. Many customers want more detailed information about goods that are on route and to be able to trace the current location of goods in real time. Some customers also want transportation services for special goods at low costs. The above research results laid a foundation for research on the location and positioning of intelligent logistics management systems. However, most research results focus on the concept of, and improvement to, blockchain technology in logistics. Based on IoT and blockchain technology, this paper proposes an optimal warehouse location method for intelligent transportation. This method combines a variety of complex mechanisms, allows IoT equipment input, and promotes the development and design of logistics applications with blockchain. As a result, this method can be used to track the transportation itinerary of products during the entire supply chain process. On this basis, using the decentralized characteristics of blockchain, this method provides full support to its trust mechanism to establish a trust relationship between the demanders and the providers. Meanwhile this method can handle transaction processes more transparently. Combining with big data from an Internet database, we will broaden the practical applications and fully establish a divergent and large-scale Internet of Things system.

## 3. Statement of Warehouse Management System Based on IoT

### 3.1. Radio Frequency Identification Indoor Positioning Technology

Radio frequency identification indoor positioning technology uses radio frequencies to modulate radio signals into electromagnetic fields and then attach them to objects, so that the magnetic fields can induce currents and transmit data and information, obtain the specific position of objects for the purpose of triangulating communications, and exchange data [25].

### 3.2. WIFI Indoor Positioning Technology

Wireless fidelity (WIFI) positioning technology aims to access wireless signals sent by terminal equipment and different third-party wireless devices, and then analyze the signal strength of a radio using a differential algorithm, using a three-point positioning method for accurate positioning [4]. WIFI positioning technology is most suitable for positioning and navigation of people or cars using intelligent mobile devices, and it can also be used in factories, shopping malls, hospitals, and other application scenarios that require positioning and guidance.

### 3.3. Infrared Positioning Technology

The principle of locating indoor objects is to emit infrared rays from emitting equipment, receive infrared rays using optical sensors installed indoors, and then locate objects by analyzing the propagation path of the infrared rays [26]. Although infrared rays can be accurately located indoors, a characteristic of light is that it cannot penetrate walls, and most opaque materials result in a limited linear propagation distance for infrared rays. In an environment with light and obstacles, the positioning accuracy of infrared rays will be affected, and the cost of installing multiple infrared receivers indoors will increase. Therefore, application scenarios for infrared positioning technology are mostly over short distances, and the influence of indoor or external light also causes infrared positioning technology to have limitations.

### 3.4. Bluetooth Positioning Technology

Bluetooth positioning technology also uses a radio for positioning, and the power consumption of this positioning technology is extremely low. By installing a connection port for a Bluetooth LAN in a room, the network mode is set to a network connection mode that can be used by many people. Bluetooth connection ports that are accessed by all users are guaranteed to be connected to the same host device, so that the location information of all users connected to the host Bluetooth device can be obtained through the Bluetooth host device [27]. The biggest advantage of Bluetooth indoor positioning technology is its small hardware size, which is easy to integrate in mobile terminals and PCs, and is as easy to popularize as WIFI.

## 4. Research Method

### 4.1. Construction of Theoretical Framework

#### 4.1.1. IoT Positioning Technology

The technologies used to obtain the position of objects using IoT are collectively called “positioning technology”. In IoT, the accurate positioning of people or things is the basis of IoT implementation. In early navigation technology, ordinary ships could only sail according to navigation lines and with pilot lights. This positioning technology had a low efficiency, large error margins and a small coverage area; it also needed manual intervention, which consumed significant manpower, material resources, and financial resources. Early IoT positioning technology was used for issuing commands on a battlefield, knowing the position of troops, and making strategic adjustments and ordering precise strikes. Presently, IoT positioning technology is mostly used in intelligent transportation and disaster relief. In order to effectively navigate and track vehicles in intelligent transportation systems, a satellite navigation system is needed to determine the location information of vehicles in real time. With the progress and development of human society, the birth of radio technology and satellites have brought about more effective positioning methods. Using radio and satellites for positioning can allow a greater positioning range and a higher accuracy. With the Wenchuan earthquake relief efforts, the Beidou communication system could quickly and accurately locate survivors to facilitate rescue. The Beidou satellite was used to scan for vital signs in a wide range, and quickly transmitted the information back to the command center. Regardless of the aspect or field in the data age, IoT positioning technology of has played a key role.

**Figure 1:**
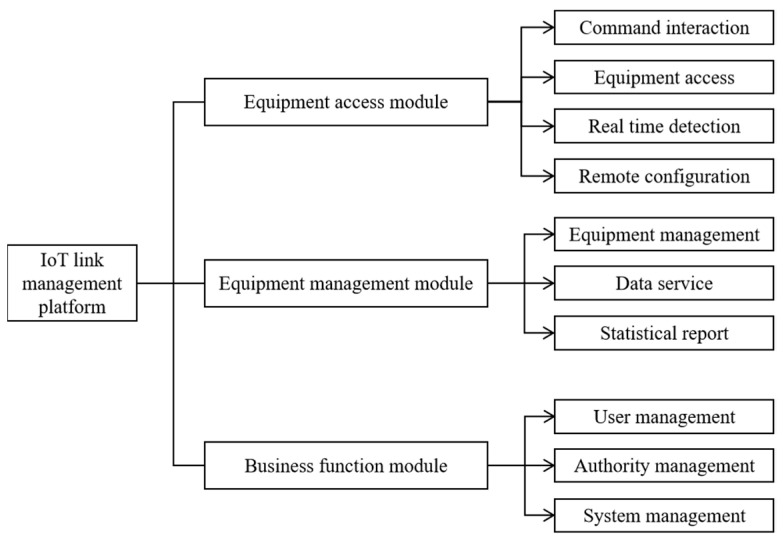
Overall architecture of an IoT connection management platform.

Based on the traditional logistics industry, applying the latest computer technology to logistics systems makes the operational process of logistics enterprises simpler and more efficient. It also allows users to have a richer and more human experience, and supports the special transportation of special goods, which is a new trend in current logistics development. The overall architecture of an IoT connection management platform is shown in Figure 1.

Environmental sensing equipment can intelligently adjust cold, hot, wet or dry environments in a warehouse by sensing indoor and outdoor temperatures and humidity, which is more conducive to the proper storage of goods. With the development of the information era, the amount of data is increasing day by day, which requires greater efficiency and accuracy in terms of data collection, which involves the hierarchical processing of data by equipment. Multi-functional nodes need to complete physical positioning, information identification, dynamic collection and pre-processing of over-limit warnings, and, finally, integrate warehouse operation information in a computer monitoring and information processing center.

#### 4.1.2. Blockchain Promotes IoT Application in Intelligent Logistics

Blockchain can realize comprehensive transmission of information over an entire network and check the accuracy of information throughout the hierarchical structure of a scattered network connected by nodes. This feature improves the convenience and intelligence of IoT transactions to a certain extent. Blockchain points have free access, and can independently participate in, or leave, the blockchain system without any causing any interference to the whole system. Based on in-depth research and applications of blockchain, it is more closely integrated with big data. In the future, more solutions can be derived from blockchain + big data solutions, and IoT will be improved at the infrastructure level.

**Figure 2:**
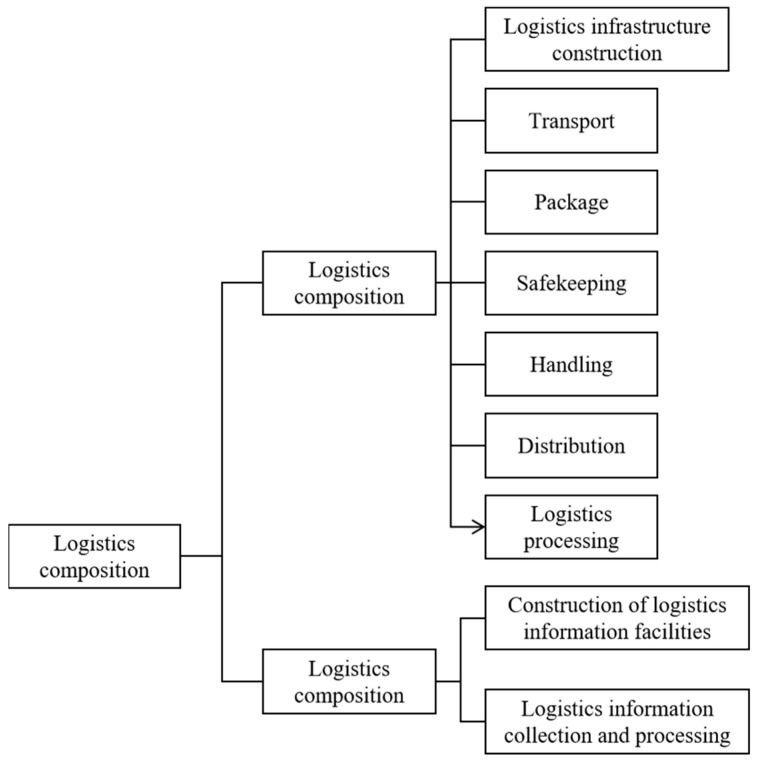
The basic elements of logistics.

“Modern logistics” refers to an integrated management system. It is a combination of logistic activities, such as transportation, information, inventory, storage, packaging, and processing. The elements of modern logistics are shown in Figure 2.

**Figure 3:**
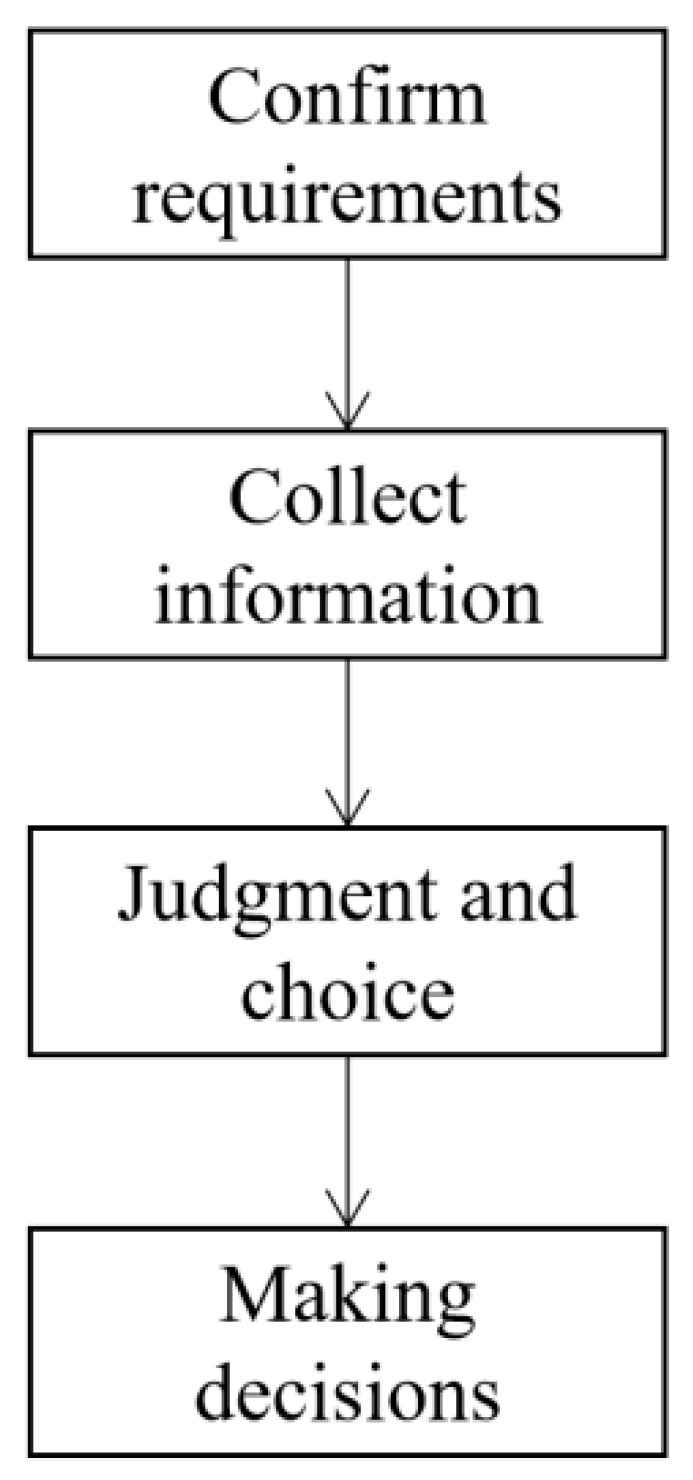
The decision-making process of IoT users.

Blockchain + big data solutions make full use of the integration capabilities of big data to promote a directional expansion of basic IoT users, and also facilitate user expansion among dispersed smart logistics users. Figure 3 shows the decision-making process of the logistics for Internet of Vehicles users.

**Figure 4:**
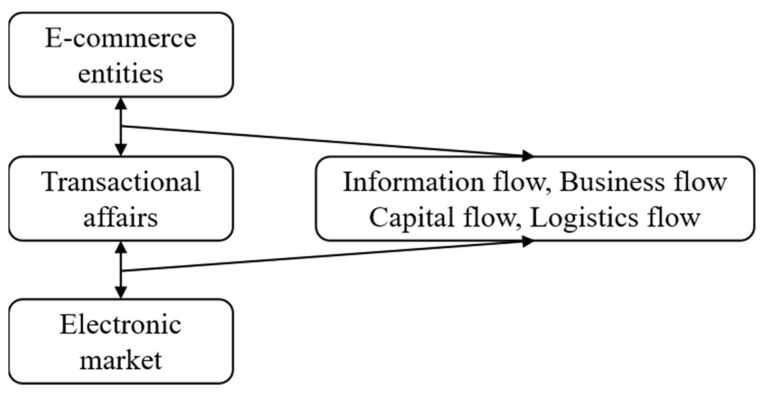
Blockchain-based IoT model and conceptual model of an urban logistics system.

Blockchain + big data solutions utilize the automatic filtering mode of big data, establishing credit resources in the blockchain, which improves the security of transactions and the convenience of IoT transactions. An IoT model based on blockchain and the conceptual model of an urban logistics system are shown in Figure 4.

The emergence of IoT technology has increased convenience in human society, and as people rely on food, such as fruits and vegetables, as well as medicines and other items, special storage conditions are needed. When storing dangerous and corrosive goods, a warehouse management system should use the sensing functions of IoT positioning technology to understand the security of the storage environment and to control risks related to changes in environment in real time. In transportation and distribution, real-time and accurate calculations are also needed to greatly improve work efficiency. In the era of big data, using IoT technology can provide more optimized configuration for warehouse management; it can reduce social logistics costs and achieve more efficient service goals.

### 4.2. Empirical Analysis

**Figure 5:**
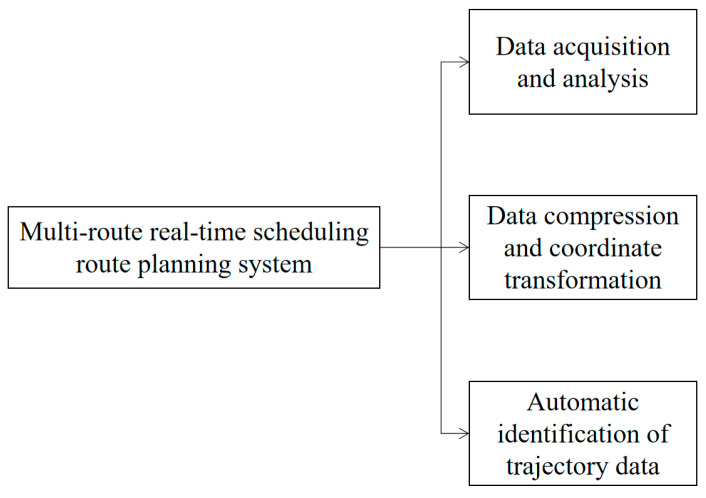
Multi-route real-time scheduling planning model.

Warehouse location is essentially a planning problem. For planning problems, optimization methods are usually used to minimize costs or maximize profits. The problem of intelligent transportation logistics in the context of an optimal warehouse location is how to locate the optimal warehouse, to ensure the lowest cost of transporting goods from the warehouse to each demand point. A multi-route real-time scheduling model for intelligent transportation logistics is shown in Figure 5.

Among heuristic algorithms, the tabu search algorithm has a global optimization ability and it is easy to implement; however, its local search performance is easily affected by dispersion. The ant colony algorithm introduces a positive feedback parallel mechanism, which has the advantages of a strong robustness, excellent distributed computing mechanism, and it can be easily combined with other methods. However, its global optimization performance is largely related to the selection of the evaporation coefficient. If this is not properly selected, the algorithm will easily fall into a local optimum. Therefore, this paper combines the two methods to make up for their respective shortcomings. As only some road sections change with a change in warehouse location, this research can be limited to those road sections. Therefore, the vehicle routing stage is actually local research, rather than global research in terms of moving all routes, which will eliminate many unnecessary calculations and allow the two-stage algorithm to obtain a better solution with a reasonable calculation time.

Suppose there are *n* warehouses for a certain item, and there are m demand points that require this item. Suppose also that the quantity of items that each warehouse can provide is $q_{1},q_{2},\ldots ,q_{n}$, the demand for each demand point is $g_{1},g_{2},\ldots ,g_{m}$, and the unit commodity transportation cost from warehouse *i* to demand point *j* is $w_{ij}$ yuan. Transform the transportation problem into a linear programming problem. Use $P_{ij}$ to describe the number of goods transported from warehouse *i* to demand point *j*, where i=1,2,…,n, j=1,2,…,m, and the number of decision variables is *n* × *m*.

Restrictions:

(1) $$\sum_{i=1}^{n}P_{ij}\leq q_{n}$$

(2) $$\sum_{j=1}^{m}P_{ij}\geq g_{m}$$

(3) $$P_{ij}\geq 0$$

Objective function:

(4) $$W_{\min}=\min\sum_{i=1}^{n}\sum_{j=1}^{m}P_{ij}w_{ij}$$

For any warehouse (*i*) and demand point (*j*), supply constraints require that the storage volume of goods in a warehouse should not be less than the shipped volume, and the total amount shipped to any demand point reaches a minimum requirement for that point. Removing non-negative constraints, there are *n* + *m* constraints. The market demand can only be met when the total warehouse supply is equal to the minimum market demand. The formula for this is described as follows:

(5) $$\sum_{i=1}^{n}q_{i}>\sum_{j=1}^{m}g_{j}$$

The total supply of a warehouse is required to be equal to the minimum total demand, namely:

(6) $$\sum_{i=1}^{n}q_{i}=\sum_{j=1}^{m}g_{j}$$

All items that can be supplied by the warehouse are transported to the demand point to meet the minimum demand of the market. In this case, all supply and demand constraints are described in an equation form, which can be translated into the following standard transportation problem.

Restrictions:

(7) $$\sum_{i=1}^{n}P_{ij}\leq q_{n}$$

(8) $$\sum_{j=1}^{m}P_{ij}\geq g_{m}$$

(9) $$P_{ij}\geq 0$$

Objective function:

(10) $$W_{\min}=\min\sum_{i=1}^{n}\sum_{j=1}^{m}P_{ij}w_{ij}$$

Since the tabu search algorithm always starts with the initial solution, it is necessary to calculate the initial solution before calculation. The tabu search algorithm has a strong dependence on the initial solution. A good initial solution can allow tabu search to find a better solution in the solution space, while a poor initial solution will reduce the convergence speed and search quality. Therefore, a greedy heuristic algorithm is used to get a better initial solution to improve the performance of the algorithm. This research uses *S* to describe the set of s candidate warehouses in terms of intelligent transportation logistics, *U* to describe the selected point set, $S\left(U\right)=\left\{U_{1},U_{2},\ldots ,U_{v\left(n-v\right)}\right\}$ to represent the corresponding field, *v* to describe the number of locating warehouses, and tabu_tag (r) to describe the taboo state of node r. Then, the objective function solving process is as follows:

(1) Obtain the initial solution through the greedy heuristic method.
(2) Input parameters, including the number of termination iteration steps, δ*. During iteration, the number of neighbors (K), taboo length h and the penalty function of the current solution are solved. Let δ=0, tabu_tag(r)=0, A=φ, to acquire the best solution, $U_{best}$, at that moment, let $U_{best}=U$, and the evaluation value *E* of *U* should be found.
(3) The current optimal solution, *U,* is processed using the pairwise exchange method. If the exchange point is not an element in taboo table A, a certain area, *U*′, of *U* can be obtained, and the solution to *U*′ can be obtained using the solution evaluation method. The evaluation of value *E*′ is performed, obtaining a path using the ant colony method, and the taboo table is updated.
(4) Judge whether the process reaches the number of termination iteration steps, δ*. If it reaches this value, output the results; otherwise, repeat step (3).

## 5. Result Analysis and Discussion

The accurate location of a warehouse is the basis of the optimal location in intelligent transportation logistics. Firstly, the accuracy of the Beidou location needs to be verified, and then whether the optimal location results achieve the low-cost target needs to be verified. $x_{i}\in R^{n}$ is a factor that affects the forecast of the logistics situation and $y_{i}$ is the forecast value of the logistics situation. Determining the optimal warehouse positioning of intelligent transportation logistics based on blockchain is to find the relationship between $x_{i}$ and $y_{i}$:

(11) $$f:R^{n}\to R$$

(12) $$y_{i}=f\left(x_{i}\right)$$

In the formula, $R^{n}$ is a factor that affects the location of the warehouse. The establishment of the logistics status prediction model seeks to establish the following expression:

(13) $$f\left(x\right)=\sum_{i=1}^{k}\left(a_{i}-a_{i}^{*}\right)K\left(x,x_{i}\right)+b$$

In the formula: x is the factor that affects the logistics status, $x_{i}$ is the i sample among k samples, and $K\left(x,x_{i}\right)$ is the kernel function. The kernel function adopts the radial basis function, as shown in the following formula:

(14) $$K\left(x,y\right)=\exp\left|-\frac{{\left\|x-y\right\|}^{2}}{2\sigma^{2}}\right|$$

**Figure 6:**
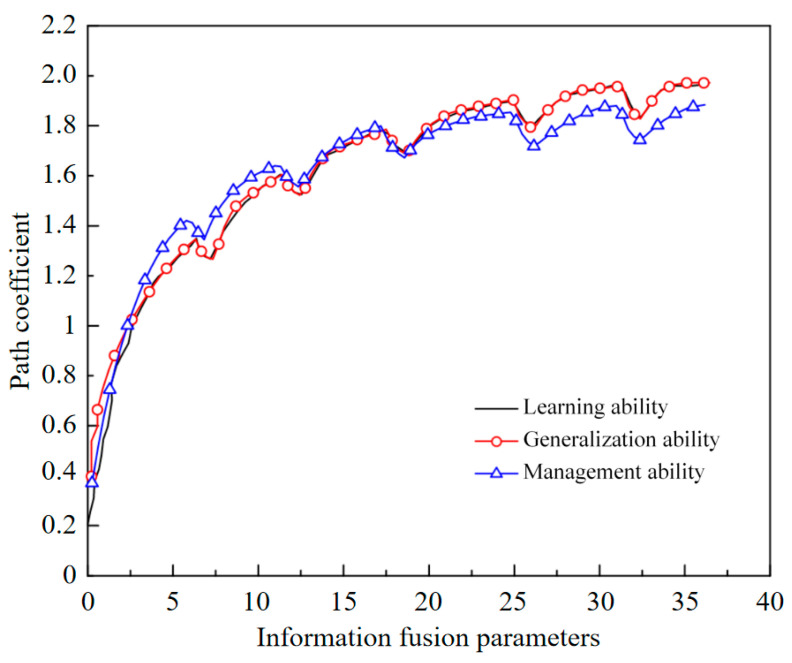
The relationship between information fusion parameters and paths.

Spatial information fusion is the multi-level and multi-faceted detection, correlation, correlation, estimation, and synthesis of data and information from multiple sensors. As shown in Table 1, it is a parameter estimation and significance test of the spatial information fusion index structure. The relationship between the information fusion parameter value and the path is shown in Figure 6.

**Figure 7:**
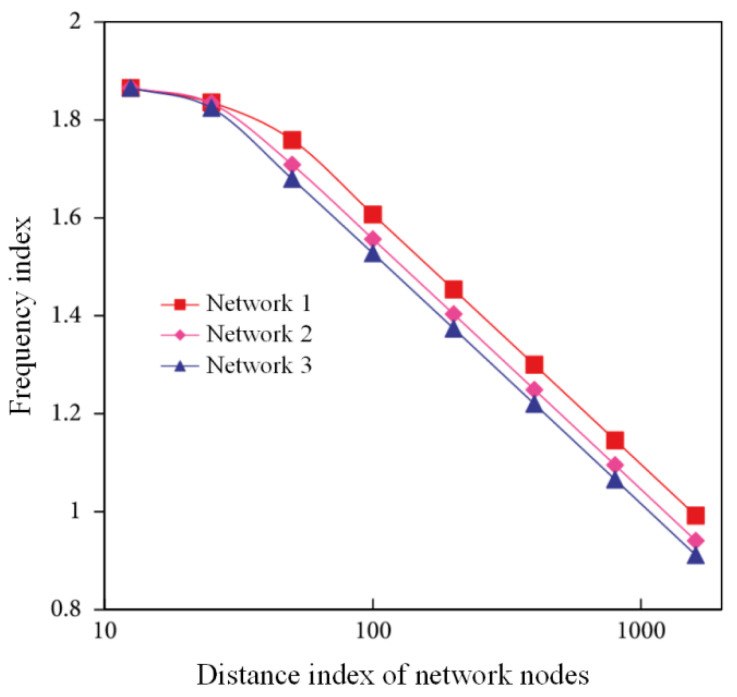
Distance analysis of nodes in intelligent transportation logistics network.

**Figure 8:**
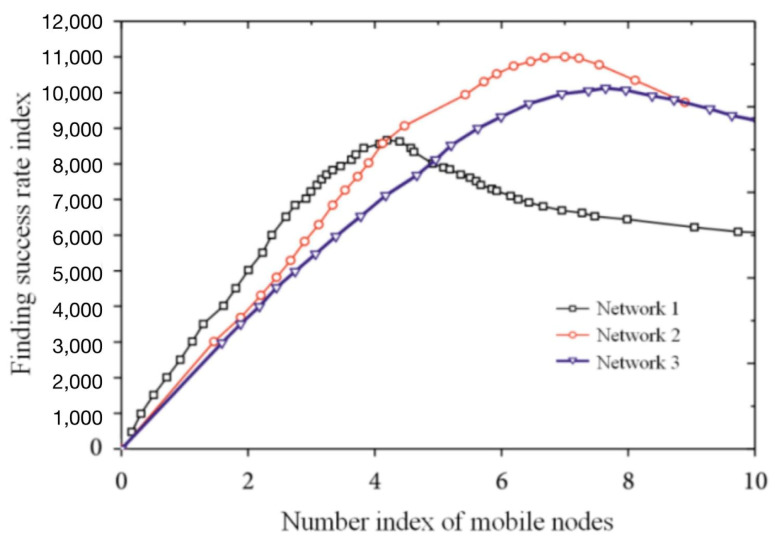
Perceived service node search success rate.

The wood of a wireless sensor network in IoT has the characteristics of a dynamic topology, limited energy consumption, limited node resources, unreliable data transmission, etc. It is necessary to improve the system to meet the performance requirements of a wireless sensor network for a task scheduling system from four aspects: practicality, economy, energy saving, and coordination. Analyzing the number of social relations, network density, and central potential of intelligent transportation logistics network should be done, as shown in Table 2. With an increase in the social relations between nodes, the shortest path between nodes and the average distance of the whole network are decreased, as shown in Figure 7. See Figure 8 for data regarding the search success rate of the perceived service nodes.

**Figure 9:**
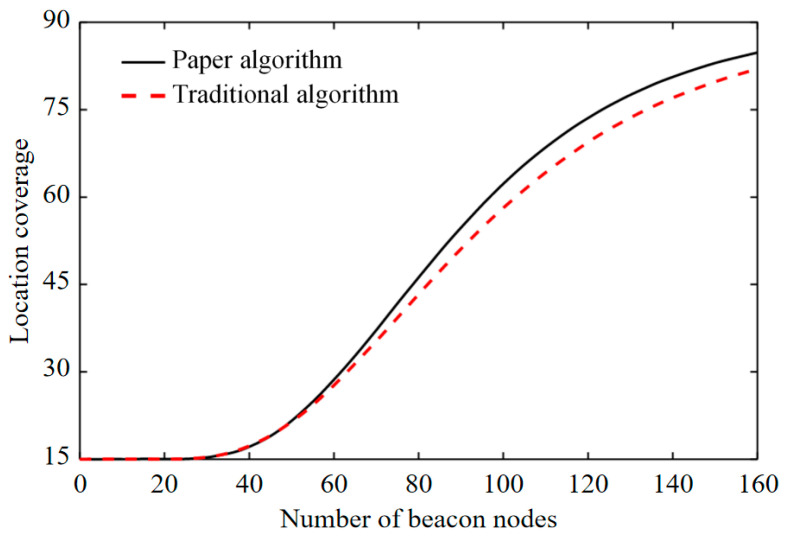
Location coverage.

Through empirical results, it can be determined that IoT and blockchain technology have a good application effect in intelligent transportation and warehouse positioning. In the scheduling process, vehicles are first scheduled according to current traffic volumes and priority, then the vehicles can start moving after being fully loaded, according to the priority order. When there is no vehicle on site, after the arrival of high priority business, or when vehicles that are on site cannot meet the transportation demands of the current business, the current lowest priority running business will be stopped, and the goods at the transfer station nearest to the current driving point will be unloaded. In the process of cargo transportation, if there are special environmental requirements, special cargo transportation vehicles can be adopted and the on-board equipment carried by such vehicles can integrate various sensors. When the goods are received, the alarm threshold of each sensor is set, and the collected data are sent back to the server in real time via GPRS for subsequent processing. When logistics personnel use cargo transport vehicles to work in the logistics warehouses, they will transport the goods to specific cargo positions so as to realize centralized placement of the goods, which is convenient for later management and pickup. As shown in Figure 9, with an increase in the number of beacon nodes, the positioning coverage of the improved algorithm is significantly greater than that of the traditional positioning algorithm.

When using background management with the system, the logistics operators can inquire about relevant department information, relevant employee information, and announcement message content for the company, as well as manage basic report data information and inquire about the basic data of related reports. In the scheduling process, first, vehicles are scheduled according to current traffic volumes and priority, and then the vehicles can start moving after being fully loaded according to the priority order. When there is no vehicle on site after the arrival of high priority business, or when the vehicles that are on site cannot meet the transportation demands of the current business, the currently running business with lowest priority is stopped and goods are unload at the transfer station nearest to the current driving point.

## 6. Conclusions and Future Work

For traditional logistics and warehousing operations, the application of IoT positioning technology has significantly increased the efficiency and stability of the industry, and can adapt to the service needs of today’s society. Blockchain is flexible and open, and based on these attributes, the industrialization of blockchain is bound to show progress. Based on the ideas of big data and internet communication, IoT technology is being used in warehouse management systems more widely. IoT provides convenience for warehouse management systems, but it also faces great challenges in terms of cost control and network security. This paper proposes an optimal location method of intelligent transportation logistics warehouse based on Internet of Things blockchain technology. This application combines a variety of complex mechanisms and the design of blockchain logistics. Therefore, this application sets up efficient and accurate tracking and traceability control throughout the entire supply chain process.

In this paper, the objective function is established and solved using the tabu search method, and the optimal warehouse location result is obtained. The experimental results show that the proposed method has a high overall performance. The location method of intelligent transportation logistics warehouse perfectly handles strategic processes and caters to the trends of modern enterprises. With input from IoT devices, many different functions have been successfully realized. Based on the analysis of current logistics systems and according to the continuous developments in the logistics industry, an intelligent logistics system based on IoT and blockchain technology is proposed. In the process of transportation, if there are special requirements, special vehicles can be used, and the on-board equipment of such vehicles can integrate various sensors. When the goods are received, alarm thresholds are set for each sensor and the collected data are sent back to the server in real time via GPRS. According to this design scheme, real-time querying of goods-in-transit information can be realized, and work efficiency can be improved. Although blockchain technology has already been used to support complex applications, the application of these systems is still limited. The most typical bottlenecks are scalability management and the high storage costs. The blockchain + big data model is still in a stage of continuous development and exploration. Therefore, there is still a long way to go in terms of the development process of this combination. The application of intelligent logistics may be further developed and applied with the continuous improvement of the blockchain + big data model in the future.

## Figures and Tables

**Table 1:** Information fusion parameter estimation and significance test.

Path Description	Fusion Parameters	Path Coefficient
Learning ability	6.68	6.76
Generalization ability	5.24	5.21
Management ability	6.35	5.55

**Table 2:** Structure analysis of intelligent transportation logistics network.

Network	Number of Nodes	Number of Relationships	Network Density	Central Potential
Meet information	165	215	0.745	0.231
Mutual information	177	233	0.568	0.237
Weighted summation	161	221	0.561	0.231

## Data Availability

Relevant data are available upon request to all authors.
